# Immune regulation and therapeutic application of T regulatory cells in liver diseases

**DOI:** 10.3389/fimmu.2024.1371089

**Published:** 2024-03-20

**Authors:** Ananya Ajith, Makram Merimi, Mandana Kazem Arki, Nikoo Hossein-khannazer, Mehdi Najar, Massoud Vosough, Etienne Marc Sokal, Mustapha Najimi

**Affiliations:** ^1^ Laboratory of Pediatric Hepatology and Cell Therapy, Institute of Experimental and Clinical Research (IREC), UCLouvain, Brussels, Belgium; ^2^ Genetics and Immune Cell Therapy Unit, LBBES Laboratory, Faculty of Sciences, University Mohammed Premier, Oujda, Morocco; ^3^ Gastroenterology and Liver Diseases Research Center, Research Institute for Gastroenterology and Liver Diseases, Shahid Beheshti University of Medical Sciences, Tehran, Iran; ^4^ Department of Tissue Engineering and Applied Cell Sciences, School of Advanced Technologies in Medicine, Shahid Beheshti University of Medical Sciences, Tehran, Iran; ^5^ Osteoarthritis Research Unit, Department of Medicine, University of Montreal Hospital Research Center (CRCHUM), Montreal, QC, Canada; ^6^ Faculty of Medicine, Université Libre de Bruxelles, Brussels, Belgium; ^7^ Department of Regenerative Medicine, Cell Science Research Centre, Royan Institute for Stem Cell Biology and Technology, ACECR, Tehran, Iran; ^8^ Experimental Cancer Medicine, Institution for Laboratory Medicine, Karolinska Institute, Huddinge, Sweden

**Keywords:** T regulatory cells, foxp3, hepatic microenvironment, liver fibrosis, liver cirrhosis, HCC, Treg immunotherapy

## Abstract

CD4^+^ CD25^+^ FOXP3^+^ T regulatory cells (Tregs) are a subset of the immunomodulatory cell population that can inhibit both innate and adaptive immunity by various regulatory mechanisms. In hepatic microenvironment, proliferation, plasticity, migration, and function of Tregs are interrelated to the remaining immune cells and their secreted cytokines and chemokines. In normal conditions, Tregs protect the liver from inflammatory and auto-immune responses, while disruption of this crosstalk between Tregs and other immune cells may result in the progression of chronic liver diseases and the development of hepatic malignancy. In this review, we analyze the deviance of this protective nature of Tregs in response to chronic inflammation and its involvement in inducing liver fibrosis, cirrhosis, and hepatocellular carcinoma. We will also provide a detailed emphasis on the relevance of Tregs as an effective immunotherapeutic option for autoimmune diseases, liver transplantation, and chronic liver diseases including liver cancer.

## Introduction

Tregs are a minor population of T subset cells, representing about 5-10% of the whole cluster of differentiation (CD) 4^+^ T lymphocyte population (1, 2). Tregs maintain immune homeostasis by controlling autoimmune reactions and imparting self-tolerance in tissues (3). In both Humans and other mammals, Tregs prevent self-antigen reactions by suppressing immune cells of innate lineages like monocytes, macrophages, dendritic cells (DCs), natural killer cells (NKs), and natural killer T cells (NKTs) as well as immune cells of adaptive lineage such as T and B lymphocytes (4, 5). Treg family comprises of natural Tregs (nTregs) that express the nuclear forkhead or winged-helix family of transcription factor P3 (FoxP3), along with cell surface proteins cytotoxic T lymphocyte antigen-4 (CTLA-4) and CD25, as well as peripherally derived Tregs (pTregs) or those generated *in vitro*, known as induced Tregs (iTregs) (6). nTregs are characterized as CD4^+^ T cells expressing high levels of CD25 and FOXP3, and low levels of CD127 surface marker (7–9). CD25 is the α-chain of Interleukin-2 (IL-2) receptor expressed on the cell surface of Tregs and activated T effector cells. Transcription factor FOXP3 is crucial for the development, function, and lineage commitment of Tregs (7, 10). It has been reported as a specific marker required for the development of thymic CD4^+^ CD25^+^ Tregs and is directly correlated with the cell surface expression of CD25 receptor (11). Mutations or deficiency of the FOXP3 gene can lead to IPEX syndrome (immune dysregulation, polyendocrinopathy, enteropathy, X-linked genetic trait) in humans, and in scurfy mutant mice, it causes lymphoproliferation and multiorgan autoimmunity (11, 12).

## Subsets of Tregs

**Figure 1:**
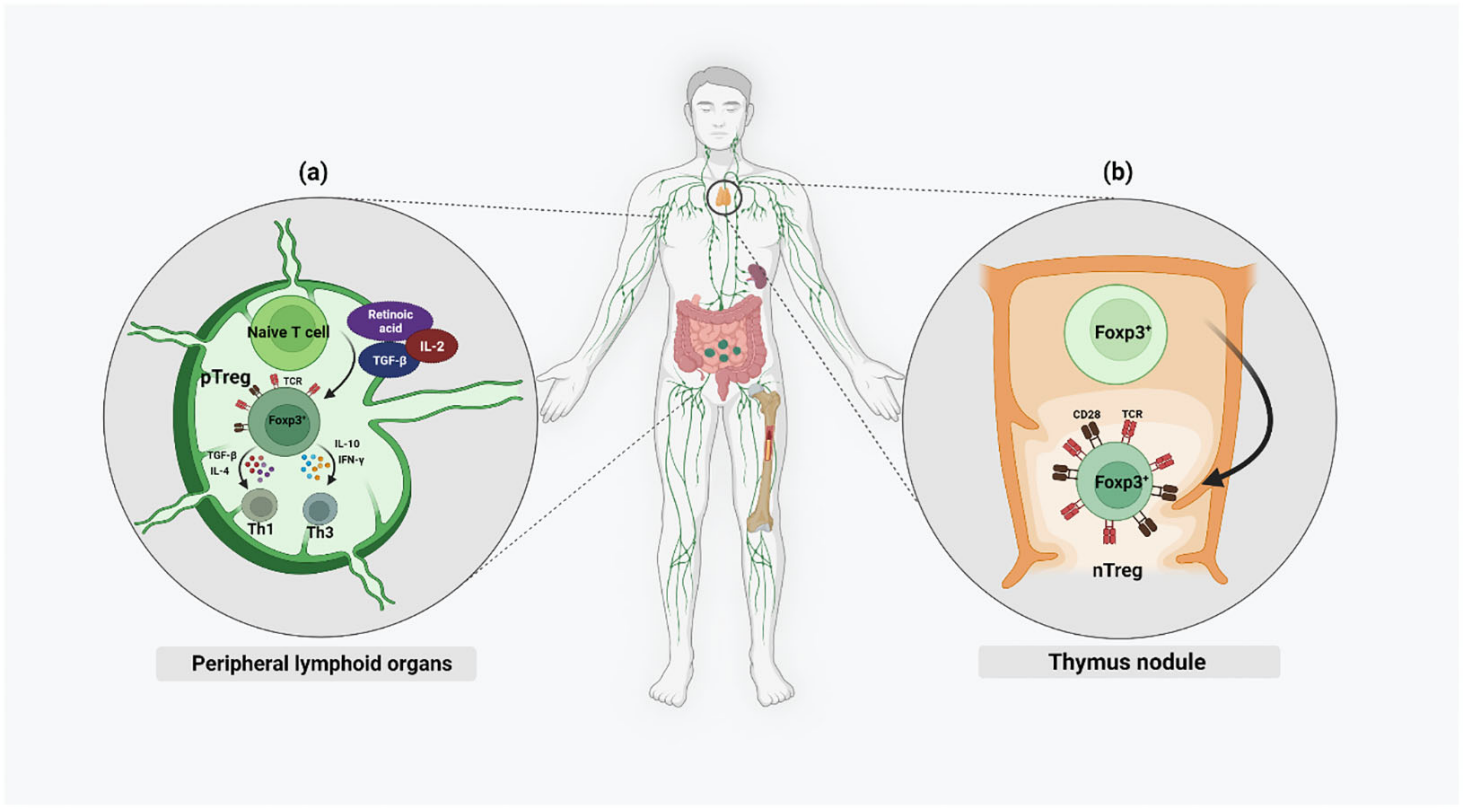
Different chemokines and cytokines activate both nTregs and pTregs in the microenvironment of both thymus and the periphery. Naïve nTregs are already FoxP3^+^ and their activation is mainly by strong TCR stimulation and CD28 co-stimulation. Only activated pTregs express FoxP3 and this is mainly by weak TCR stimulation along with higher levels of IL-2, TGF-β, and RA.

Regulatory T cells are classified into Tregs expressing Foxp3 (Foxp3^+^ Tregs), Type 1 regulatory T (Tr1) cells, T helper (Th3) cells, CD8^+^CD28^−^ T cells, human leukocyte antigen (HLA)‐G^+^CD4^+^ T cells and HLA‐E‐specific CD8^+^ T cells (13). In this review we will focus on the CD4+ Tregs. FoxP3 expressing Tregs are a highly heterogenous population of immune cells. Based on their site of development, Tregs are subdivided into naturally occurring Tregs (nTregs) and adaptive or induced Tregs (pTregs). While nTregs develop in the thymus, pTregs develop in the peripheral lymphoid organs (14) (**Figure 1**). Strong T-cell Receptor (TCR) signaling and CD28 co-stimulation result in the nTreg lineage, whereas pTregs are generated from naïve, mature T cells under weak TCR stimulation along with other factors like IL-2, Transforming growth factor-β (TGF-β) or retinoic acid (RA) (2, 15). High demethylation of Treg-specific demethylated region (TSDR), a highly conserved CpG-rich region in the FOXP3 enhancer, is specific for nTregs while in pTregs, it is only partially demethylated (2). Th-3 and Th1-like or Tr1 are Treg subsets arising from pTregs in the periphery. The presence of cytokines like TGF-β and IL-4 promotes the proliferation of Th3 Tregs while IL-10 and Interferon (IFN)-γ promote the proliferation of the Th1-like or Tr1 lineages of Treg cell types. Th-3 Tregs are FoxP3^+^ and they largely maintain the immunosuppressive microenvironment by secreting TGF-β. Th1 Tregs lack FoxP3 expression, but express Lymphocyte-activation gene 3 (LAG-3) and secrete IL-10 into their microenvironment (16).

Furthermore, Tregs can be subdivided based on their differentiation potential into naïve Tregs (nTregs), effector Tregs (eTregs), effector memory cells (emTregs), and central memory cells (cmTregs) (**Table 1**) (18). Peripheral self-antigens activate nTregs to increase their cell number and suppressive function, thereby differentiating them into eTregs, which are recruited toward the inflammation site. A proportion of these cells remain in the non-lymphoid tissue after antigen expression cessation and survive as effector memory Tregs (emTregs) with an enhanced capacity to suppress autoimmunity in these tissues (21). Apart from this, memory Tregs may be located in the secondary lymphoid organs as the central memory Tregs (cmTregs). A re-exposure to the antigen can result in an expansion of these memory Tregs into a more effective immune response than during the primary exposure (17).

**Table 1:** Site of localization and marker heterogeneity in different Treg subsets.

Cell type	Localisation	Specific Markers	References
Naïve Tregs (nTregs)	Thymus Secondary lymphoid organs	FoxP3^+^, CD25^+^, CD45RA^+^, CD45RO^-^, CCR7^+^, CD62L^+^, CTLA4^-^, CD127^-^	(17, 18)
Effector Tregs (eTregs)	Sites of inflammation	FoxP3^+^, CD25^+/-^, CD45RA^-^, CD45RO^+^, CCR7^+^, CD62L^+^, CTLA4^+^, CD127^-^	
Central memory Tregs (cmTregs)	Secondary lymphoid organs	FoxP3^+^, CD25^+^, CD45RA^-^, CD45RO^+^, CCR7^-^, CD62L^-^, CTLA4^+^, CD127^-^	
Effector memory Tregs (emTregs)	Tissue-resident	FoxP3^+^, CD25^+^, CD45RA^-^, CD45RO^+^, CCR7^-^, CD62L^-^, CTLA4^+^, CD127^+^	
Induced Tregs (iTregs)	*In vitro* culture	FoxP3^+^, CD25^+^, CTLA4^+^, GITR^+^, LAG3^+^, CD62L^+^, Helios^-^	(19, 20)

## Suppressive mechanisms of Tregs

In homeostatic conditions, Tregs show reduced recirculation or tissue infiltration in comparison to conventional T cells. During pathological conditions, Treg recirculation through the lymphoid tissues for entering the inflamed, tumors, and infectious sites, increases (22). Tregs induce immunosuppression by inhibiting the proliferation, differentiation, cytokine or antibody production, of their target cells. Tregs can suppress a large number of target immune cells by direct cell-cell contact inhibition/killing, secretion of anti-inflammatory cytokines, and disruption of the metabolic pathways competing for growth factors (23). Direct cell-cell contact elimination is a strategy used by nTregs to directly kill cytotoxic cells by releasing perforin or serine protease granzyme (24). Activated Tregs may express cell surface molecules like Galectin1 which bind the receptors of effector T cells to induce cell cycle arrest, apoptosis, or inhibition of pro-inflammatory cytokine secretions. Cell-cell contact of Tregs with target cells can also induce several inhibitory pathways by the modulation of molecules such as cyclic adenosine monophosphate (cAMP), resulting in the inhibition of cellular proliferation, differentiation, inhibition of cytokines IL-2, IFNγ or the activation of transcriptional repressor inducible cAMP early repressor (ICER). In trans-well system studies, Tregs failed to suppress effector T target cells, which suggests that it is necessary for Tregs to be in close proximity of the target cells to impart their inhibitory action (25). However, recent studies showed that the secretion of extracellular vesicles (EVs) by Tregs has emerged as a novel suppressive mechanism with the ability to modulate immunity in a cell-contact independent and targeted manner. This phenomenon has been identified in various autoimmune and cancer pathologies. Yu et al., demonstrated that Tregs-derived EVs effectively suppressed T cell proliferation in a dose-dependent manner (26). In another study by Okoye et al., Treg-derived EVs (Treg-EVs) were shown to suppress T cell-mediated responses through the transfer of packaged Let-7d miRNA to Th1 cells, leading to an inhibition of their proliferation and IFN-γ secretion (27). Aiello et al. further demonstrated that EVs derived from Tregs possessed the ability to convert T cells into regulatory cells, upon delivery of miR-503, miR-330, miR-9 (28). Naïve T cells exposed to these Treg-EVs exhibited an increased IL-10 secretion and expressed T cell immunoglobulin and mucin-domain containing-3 (Tim3), indicating the regulatory effects of these EVs on T cell behavior. Such studies collectively shed light on the immunomodulatory potential of Treg-EVs, offering insights into their therapeutic implications in immune-related disorders (29). Notably, Treg-EVs appear to exhibit a dual role, either protecting or inducing cell death, contingent upon the target cells involved. These EVs demonstrate the capability to inhibit apoptosis in myocardial cells during acute myocardial infarction, while concurrently inducing apoptosis in conventional T cells.

**Figure 2:**
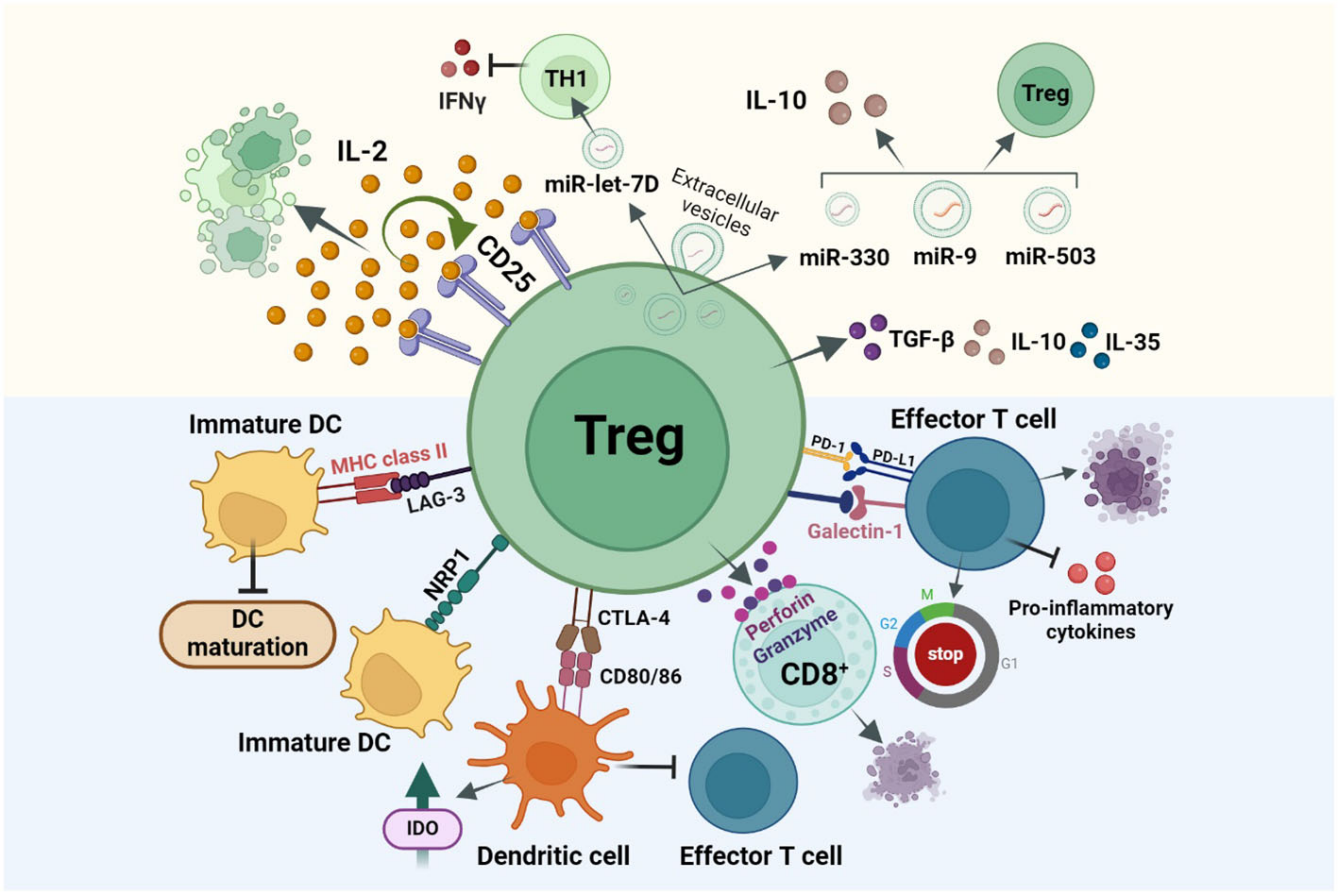
Treg immunosuppression is achieved via three main mechanisms: direct cell-cell contact inhibition or cell killing (Blue background), secretion of immunosuppressive cytokines, and disruption of metabolic pathways (Yellow background). (i) Treg cell surface molecules like, CTLA-4, LAG-3, NRP1 bind to the cell surface receptors of tolerogenic DCs resulting the DCs from maturing for antigen presentation and thereby inhibiting T effector cell activation and functioning. (ii) Secretion of immunosuppressive cytokines like IL-35, IL-10, TGF-β results in cell cycle arrest in other immune cells (iii) Tregs over-express CD25 receptor which has a higher affinity for IL-2, this in turn will suppress other cell types which require IL-2 for activation like T effector cells from effectively functioning. Other mechanisms include Ca^2+^ suppression in T effector cells, IDO based and PD-1/PDL-1 interaction-based cell suppressions.

DCs represent a major antigen-presenting cell (APC) type, so targeting this cell population can indirectly inhibit T effector cell activation and immune response. LAG-3 (CD223) expressed on Tregs, binds to the Major histocompatibility complex class II (MHC class II) in immature DCs and induces an inhibitory signal which leads to a suppression of DC maturation and their capability for immune stimulation. Prolonged interaction of Neuropilin 1 (Nrp1), present in Tregs, with immature DCs, restricts the antigen-presenting capabilities of DCs (**Figure 2**) (30, 31). Thereby, only mature DCs promote T effector cell proliferation, while immature/tolerogenic DCs induce the activity of Tregs. Thus, tolerogenic DCs-induced Tregs and the Treg-maintained immunosuppressive nature of DCs are linked (32, 33). As FOXP3 regulates the high expression of cytotoxic T lymphocyte antigen 4 (CTLA-4) required for immunosuppressive activity of Tregs (34), another strategy for DC suppression is the binding of CTLA-4 to DC surface proteins CD80/86 (**Figure 2**). Formed complex is trans-endocytosed by Tregs thereby inhibiting the ability of DCs to interact with effector T cells. Thereafter, CD80/86 is fused with the lysosome and CTLA-4 is recycled back to the cell surface for further APC suppression (35). Another mechanism of Treg-induced maintenance of tolerogenic DCs is by inducing the secretion of indoleamine 2,3-dioxygenase (IDO). Studies have shown that both in mice and Humans, Tregs induce DCs to produce IDO, in order to suppress the T cell response through CTLA-4 dependent mechanisms (36). Cheng et al. reported that the DCs recruited by the carcinoma-associated fibroblasts (CAFs) in hepatocellular carcinoma (HCC) get converted to IDO producing regulatory DCs which exhibits an increased ability to suppress T cell proliferation, and upregulation of Tregs through IL-6 mediated Signal transducer and activator of transcription 3 (STAT3) activation (37).

Programmed death ligand 1/programmed death receptor 1 (PDL-1/PD-1) based interaction between tumor cells and T lymphocytes, plays an important role in suppressing the cytotoxic effects of T cell immunity in the tumor microenvironment. Likewise, Tregs also express PD-1 and PDL-1 on their cell surface and have the potential to interact with the PDL-1-expressing T and B lymphocytes, DCs, macrophages, and bone marrow-derived mast cells. During cancer, PD-1 is upregulated in Tregs resulting in an increased interaction of PDL-1 in T effector cells thereby resulting in their inhibition and suppression of immune responses. While PDL-1/PD-1 suppresses the immunogenic activity of T lymphocytes, this interaction in Tregs may activate and induce their differentiation (38, 39). Tregs can also secrete suppressive cytokines like IL-10, TGF-β, and IL-35, which could induce cell cycle arrest in other immune and myeloid cells (**Figure 2**) (31). Studies show that deficiency of membrane-bound and soluble TGF-β produced by Tregs, results in T-cell mediated autoimmunity in mice and that IL-35 directly influences T cells by inhibiting their proliferation (40).

Key features of FoxP3^+^ Tregs are high expression of CTLA-4, CD25 cell surface marker and absence of IL-2 production (41). IL-2 is an important cytokine signal required for the proliferation, differentiation, survival and activity of T-helper cells, memory T cells and Tregs. Naive Tregs do not produce IL-2, but the cell surface receptor CD25 displays high affinity for IL-2. By competitive consumption, Tregs use the IL-2 produced by activated T cells and suppress them via a negative feed-back loop (34, 35). In IL-2 deprived T cells, Tregs induce apoptosis through BCL2 Associated Agonist of cell Death (Bad)/BCL-2 interacting mediator of cell death (Bim) driven caspase activation (42). Inducible co-stimulator (ICOS) is a member of CD-28/CTLA-4 family, widely expressed on activated CD4^+^ T cells and Tregs. The pathway activation occurs during the binding of ICOS with its ligand ICOSL, expressed on APCs (43). According to the expression of ICOS, Tregs can be divided into ICOS^+^ and ICOS^-^ Tregs. *In vitro* studies have proven that cytokine secretion is different in these 2 Treg subsets, i.e. ICOS^+^ Tregs tend to secrete more IL-10 and moderate amounts of TGF-β1, while ICOS^-^ Tregs play a suppressive function by secreting TGF-β1 alone. In mice, studies have shown that ICOS^+^ Tregs have better survival and superior suppressive properties as compared to ICOS^-^ subset. In a study by Tu et al., HCC patients have been shown to display a higher prevalence of tumor infiltrating ICOS^+^ Tregs. Due to higher proportion of FoxP3^+^ Tregs/CD4^+^ T cells and ICOS^+^ FoxP3^+^ Tregs/total FoxP3^+^ Tregs, their study concludes that ICOS^+^ FoxP3^+^ Tregs may be the main immunosuppressive cell type in HCC microenvironment (44–46). Another mechanism of Treg suppression is the deprivation of calcium levels in T effector cells. Calcium is essential for the proliferation of lymphocytes, their expression of activation-associated genes, production of chemokines and cytokines, and activation and differentiation of naïve T cells into effector or memory T cells. Tregs have been reported to suppress the calcium signaling in T conventional cells (47).

## Role of Tregs on liver T lymphocytes and innate immune cells

Tregs promote immune tolerance by modulating both innate and adaptive immunity (48). CD4^+^ CD25^+^ Tregs are the natural suppressor cells of the immune system. They function during early innate and adaptive immune responses even prior to antigen activation, indicating their capability to differentiate self from non-self-cells during immune responses (49). CD4^+^ CD25^+^ Tregs maintain immune tolerance by suppressing the activity of both CD8^+^ and CD4^+^ T cell populations during inflammatory responses (50, 51). To understand their interactions with CD8^+^ and CD4^+^ cells, Treg population was depleted in mouse. The study demonstrated that CD4^+^ T cell population remained unchanged suggesting other regulatory factors, while a significant increase in the CD8^+^ T population was shown (5). The liver is renowned for its exceptional regenerative potential and is recognized for its ability to sequester auto-reactivated T cells, facilitating their apoptotic clearance. In neonatal liver, the transcriptomic profile of thymus-derived Tregs was thoroughly examined, revealing that their intra-hepatic accumulation plays a crucial role in preserving self-tolerance and promoting liver maturation. However, adult mice livers harbor a reduced number of tissue-resident Tregs, exhibiting distinct phenotypic characteristics while retaining increased proliferation, which suggests a lineage instability (52). Indeed, the liver’s unique immunological characteristics, resulting from its constant exposure to foreign antigens, are essential for its proper function. However, this constant exposure also renders the liver susceptible to immune-mediated injuries, as any disruption in the delicate balance of immune responses can lead to inflammation and damage of the liver tissue. The innate immune system in the liver consists of NKs, NKTs, Kupffer cells (KCs), DCs, and mast cells. In both human and mouse, Tregs can inhibit the NK effector function. TGF-β produced by Tregs inhibits the cytolytic activity of NKs. Tregs kill NKs by releasing granzyme-B and cell-cell contact mechanism (53). In an autoimmune condition, NKTs can secrete both anti-inflammatory and pro-inflammatory cytokines and regulate the recruitment of Tregs to the inflammation site (54). Tregs exert a negative effect on the proliferation and cytokine production of NKTs while NKTs promote the proliferation and homing of Tregs in the liver (55). KCs are involved in the pathogenesis of viral hepatitis, steatohepatitis, alcoholic liver disease, intrahepatic cholestasis, organ rejection in liver transplantation and liver fibrosis. During fibrosis, KCs induce the activation of hepatic stellate cells (HSCs) into myofibroblasts which leads to an increase in collagen and proteoglycans production (56). Depletion of KCs has been shown to induce a complete inhibition of IL-10 production by Tregs suggesting an interaction of KCs with Tregs for inducing tolerance (57). Tregs have also a role in blocking the antigen presenting capabilities of DCs and these immature DCs can promote the immune tolerance by Tregs or by inactivating T cells whereas, mature DCs trigger the development of naïve CD4^+^ and CD8^+^ T cell populations (58).

## Influence of liver metabolic activity on the function, survival, and plasticity of Tregs

The human liver receives 70-80% of blood from the portal vein rich in nutrients and the other 20-30% from the hepatic artery rich in oxygen. Tregs can either directly use the metabolites present in the liver or from the environmental metabolism of DCs. The intrahepatic microenvironment which is, deprived of oxygen and enriched with microbes, inflammatory cytokines and hormones, plays a major role in the differentiation and function of Tregs (35). Activated Tregs generate energy using glucose, amino acids, fatty acids (FAs) and vitamins. *In vitro*, lipid biosynthesis, aerobic glycolysis and FAO were also used for the generation of ATP (59). FoxP3 expression in Tregs is correlated with higher mitochondrial mass and increased reactive oxygen species (ROS) production as demonstrated in both nTregs and iTregs. Oxidative phosphorylation (OXPHOS) and ROS play an important role in Treg signaling and homeostasis (60). Unlike other T cells, Tregs rely on FAs oxidation and OXPHOS for cell differentiation and function (61). Tregs proliferation is dependent on glycolysis and FAs oxidation. Upregulated insulin receptor INSR increases the glucose uptake in Tregs, required for their migration. Mammalian target of rapamycin (mTOR) complex 2 (mTORC2) regulates the Treg motility through Phosphoinositide 3 kinase- protein kinase B (PI3K-Akt) pathway and modulation of cytoskeleton reorganization by glycogen synthase kinase (GSK) activity. PI3K modulation is likely to alter cellular metabolism and FOXP3 expression (62). Metabolites like purine, tryptophan, RA, and glutamine maintain the induction of FOXP3 gene (60). Liver is enriched with metabolites like vitamin A, vitamin D, RA, etc. required for the generation and trafficking of Tregs (59). Vitamin A, C and D3 increase and stabilize the expression and activity of FOXP3 in Tregs (63).

## Epigenetic regulation of Tregs

Epigenetic modifications like DNA methylation, histone modifications, and microRNA regulations may significantly control the proliferation of Tregs, as well as development and facilitation of their suppressive properties. Epigenetic modification of its promoter, enhancer and CpG rich region, primarily through methylation and demethylation mechanisms, modulate the expression of FOXP3 in Tregs. In response to TCR signals, FOXP3 promoter gets activated consequently to the binding of transcription factors Nuclear factor of activate T cells (NFAT) and activator protein 1 (AP1). The enhancer region of FOXP3 is a TGF-β sensor with binding sites for NFAT and SMADs (64). TSDR is demethylated in Tregs while methylated in other T cells. Such TSDR demethylation determines the stability of FOXP3 expression in Tregs (65). Inhibition or mutations occurring in DNA methyl transferase 1 gene, results in hypomethylation of FOXP3 gene, thereby increasing its expression levels in Tregs (66). In HCC, lower methylation of FOXP3 promoter via DNA methyl transferase (DNMT1) is positively linked to a higher percentage of intra-tumoral Treg levels and tumor growth (64). Like DNA demethylation, histone modifications namely N terminal lysine methylation and acetylation are other important epigenetic alterations that can result in gene activation. Histone acetyl transferase (HAT) 300 is an important factor for the immune suppressive function of Tregs and pTregs induction (67). According to this study, p300 is required for Treg activity and maintenance of homeostasis both *in vivo* and *in vitro*. Acetylation of P300 has been shown to increase FOXP3 protein levels and FOXP3 mediated transcriptional repression of IL-2 production. Apart from p300, other HATs like Tat-interactive protein 60 kD (TIP60) and CREB-binding protein (CBP) are also involved in the development, activity and lineage specificity of Tregs (68).

MicroRNAs are another epigenetic regulator of several genes implicated in Tregs polarization and play an important role in their thymic development and function (**Table 2**) (65). miR-155 is an important epigenetic factor required for an enhanced responsiveness of Tregs to IL-2. miR-155 and miR-21 are positive enhancers of the FOXP3 activity in Tregs (76). Other miRNAs like miR-17a, miR-18a, miR-19a, miR-20a, miR19b and miR-92-1, miR-31 are negatively regulating Tregs (66). In HCC patients, the modulated expression of several miRNAs hsa-miR-182-5p, hsa-miR-214-3p, hsa-miR-129-5p and hsa-miR-30b-5p in Tregs, is targeting several signaling pathways including cytokine, chemotaxis and adhesion, thereby supporting the critical role of epigenetic regulation of this immune cell population.

**Table 2:** miRNAs critically involved in Treg cell regulation.

MicroRNA identity	Positive/Negative regulation of Tregs	Function	References
miR155	+	Treg development, proliferation and survival Upregulation of IL-2 expression	(66, 69)
miR146a	+	Induced Treg-mediated suppression of IFN-γ dependent Th1 response and inflammation	(70)
miR126	+	Suppressive activity of Tregs through PI3K/AKT pathway	(71)
miR10a	+	Attenuates Tregs conversion to follicular helper T cell lineage	(72)
miR24	–	Binding to the potential target site of 3’ UTR of FOXP3 mRNA	(73)
miR31	–	Binds to the potential target site of 3’ UTR of FOXP3 mRNA	(66)
miR15a/16	–	Reduces the expression of FOxP3 and CTLA4 Attenuates Tregs immunosuppressive activity	(74)
miR-142-3p	–	Impairs the suppressive function of Tregs by restricting the generation of cAMP needed for their immunomodulatory effect on T cells	(75)

## Tregs in chronic liver diseases

Chronic wound healing process in response to sustained inflammation is a trigger for liver fibrosis and development of cirrhosis (77). Chronic alcohol consumption, non-alcoholic steatohepatitis (NASH), viral infections like hepatitis B (HBV) and C (HCV), autoimmune hepatitis (AIH), non-alcoholic fatty liver disease (NAFLD), cholestatic liver diseases, result in the inflammatory response causing fibrosis (78, 79). There is an increased risk for NASH to progress into liver fibrosis, cirrhosis and then to HCC. In NAFLD mouse model, the expression of FoxP3 mRNA is low in liver, indicating a decreased activity of Tregs. This has a deleterious effect resulting in NAFLD progression, resulting in the imbalance of Treg/Th17 cell levels due to increased inflammatory response by Th17 cells (80). A clinical study in children revealed an association between pronounced hepatic inflammation due to NAFLD and elevated number of Foxp3^+^ lymphocytes within the lobules. Conversely, adults showcased a decline in Foxp3^+^ lymphocytes alongside increased IL-17A+ lymphocytes in the portal/periportal tracts (81). Steatohepatitis was induced in mice fed with high fat diet and treated with endotoxin, resulted in Treg deficiency, while adaptive transfer of Tregs to these mice showed reduced disease progression and liver injury (82). The impact of Tregs on NASH remains unclear while an increased count of intrahepatic Tregs was reported in a related mouse model. Surprisingly, attempts at adoptive Treg transfer or anti-CD3 therapy yielded an unforeseen outcome escalated steatosis and elevated alanine aminotransferase levels—without any other discernible impact on NASH (83). In alcoholic steatohepatitis, alcoholic hepatitis and autoimmune liver diseases like primary biliary cirrhosis and autoimmune hepatitis, the population of Tregs infiltrating the liver is higher than the circulating one (84). In this setting, sustained inflammation induces necrosis and apoptosis of hepatocytes, therefore the anti-inflammatory function of Tregs has a protective effect against the inflammatory liver responses (85). Tregs have been reported to support the progression of liver fibrosis by targeting KCs through the TGF-β pathway. Furthermore, HSCs can selectively induce Tregs by the production of RA or in an IL-2 dependent manner (86). However, IL-10 released by Tregs has been reported to inhibit the ECM production by HSCs (87, 88).

Tregs are essential for wound healing and cessation of inflammation. Yet, studies report that reduction in Treg numbers can promote fibrosis regression. Activated HSCs secrete IL-2, resulting in the abundance of Tregs in fibrotic tissue. In healthy liver, matrix metalloproteinases (MMPs) released by KCs degrade and regulate the liver ECM. TGFβ secreted by Tregs can repress the MMP secretion by KCs which may disrupt the fibrosis regression (89). Study by (79) demonstrated that depletion of Tregs was associated with liver fibrosis progression in DEREG transgenic (DEpletion of REGulatory T cells) mice. This was mainly due to an increase of CD8+ and IL-17A+ T cell populations and enhanced secretion of pro-inflammatory cytokines and chemokines (Roh et al, 2015).

TGFβ is an important cytokine required for the determination of the differentiation of Tregs and Th17 cells. Indeed, low concentrations of TGFβ synergized with IL-6, induce Th-17 cell differentiation, while high concentrations of TGFβ induce Treg differentiation from the naïve CD4^+^ T cell precursors. During liver fibrosis, secretion of higher levels of IL-6 and TGFβ occurs which in turn activates the HSCs to produce ECM proteins, leading to increased Th-17 cells and an imbalance in the Th17/Treg ratio (78).

During viral infection, immune cells release cytokines needed for the termination of infection and the eradication of infected cells. Tregs regulate a delicate balance by preventing the immune cells from destroying non-infected self-cells. Studies have shown that during HBV infection, the number of CD4^+^ CD25^+^ Tregs increases in the liver and peripheral blood as compared to normal conditions (90). The analysis of Tregs in acute, chronic and chronic severe HBV infections, showed that their numbers were higher in chronic severe HBV infection than in acute or chronic HBV infections (91). HBV induced liver fibrosis also showed a similar trend where the number of Tregs was increased in advanced HBV related liver fibrosis than in early HBV-fibrosis (87). Persistent HBV infection has an increased risk for the progression of chronic hepatitis B (CHB) to hepatic cirrhosis and HCC. The role of Tregs played during HBV-induced fibrosis is dependent on disease stage at which they are activated. If the Tregs get activated by the HBV antigen in earlier stage, they might protect the HBV from host immune response, while if the host elicited Treg response occurs in the later stage of infection, Tregs will protect against excessive inflammation and prevent further liver injury. However, for progression into fibrosis from CHB infection, immune tolerance should decrease, and inflammatory response ratio should increase (92). Frequencies of Th-17 cells, IL-22 and IL-17A were found to increase with the severity of infection while the frequency of Tregs decreased in HBV associated cirrhosis than in CHB or HCC cases. IL-17 and IL-22 activate the proliferation of HSCs and the progression of fibrosis through PI3K/AKT pathway (93). When the immune suppressive properties of Tregs in the presence of HCV inoculum were studied, a significant increase of the of CD4, CD25, FOXP3 expression and inhibitory markers like CTLA-4, LAG-3, was noticed in parallel to a decreased expression of CD127. This confirms that Treg population increases with the number of HBV antigens (94). Treg immunosuppressive activity in HBV and HCV infection has dual consequences on the disease prognosis by i) restraining the CD8^+^ and CD4^+^ T cell activity which is beneficial for the host since it prevents the inflammatory liver damage and ii) persisting the infection since it provides protection for the virus from the immunogenic activities of CD8^+^ and CD4^+^ T cells (95, 96).

## Recruitment and homing of Tregs to the inflamed liver

The ability of Tregs to migrate to the site of inflammation is critical for their capacity to regulate inflammation, and this is mainly achieved by the expression of adhesion molecules and chemokine receptors. Like conventional T cells, Tregs also undergo trans-endothelial migration by interacting with pro-adhesive signals. In general, during their migration when ligands bind to the receptors, leukocytes alter their cell surface integrin conformation (like β2 and α4 families). This allows the adhesion molecules to bind to the endothelial cell expressed ligands like intracellular adhesion molecule-1,2,3 (ICAM) and Vascular cell adhesion molecule-1 (VCAM)/mucosal addressin cell adhesion mol-1 (MAdCAM-1). This interaction with the adhesion molecules, enables the arrest of rolling leukocytes, resulting in the trans-endothelial migration of leukocytes (97). Similarly, interaction between liver sinusoidal endothelial cells (LSECs) and Tregs, leads to the activation of integrins lymphocyte function associated antigen 1 (LFA-1) and very late antigen 1 (VLA-4) on Treg surface thereby interacting with the cell adhesion molecules like intercellular adhesion molecule 1 (ICAM), vascular cell adhesion molecule (VACM) and Vascular adhesion protein-1 (VAP-1) expressed by inflamed LSECs and resulting in the trans-endothelial migration of Tregs into liver (98). In inflamed liver, Shetty and colleagues demonstrated that the homing of Tregs is also mediated by their interaction with the common lymphatic endothelial and vascular endothelial receptor (CLEVER-1) expressed by LSECs. ICAM-1 and VAP-1 along with common lymphatic endothelial and vascular endothelial receptor-1 (CLEVER-1) support the transcellular migration of Tregs in the liver (99).

**Figure 3:**
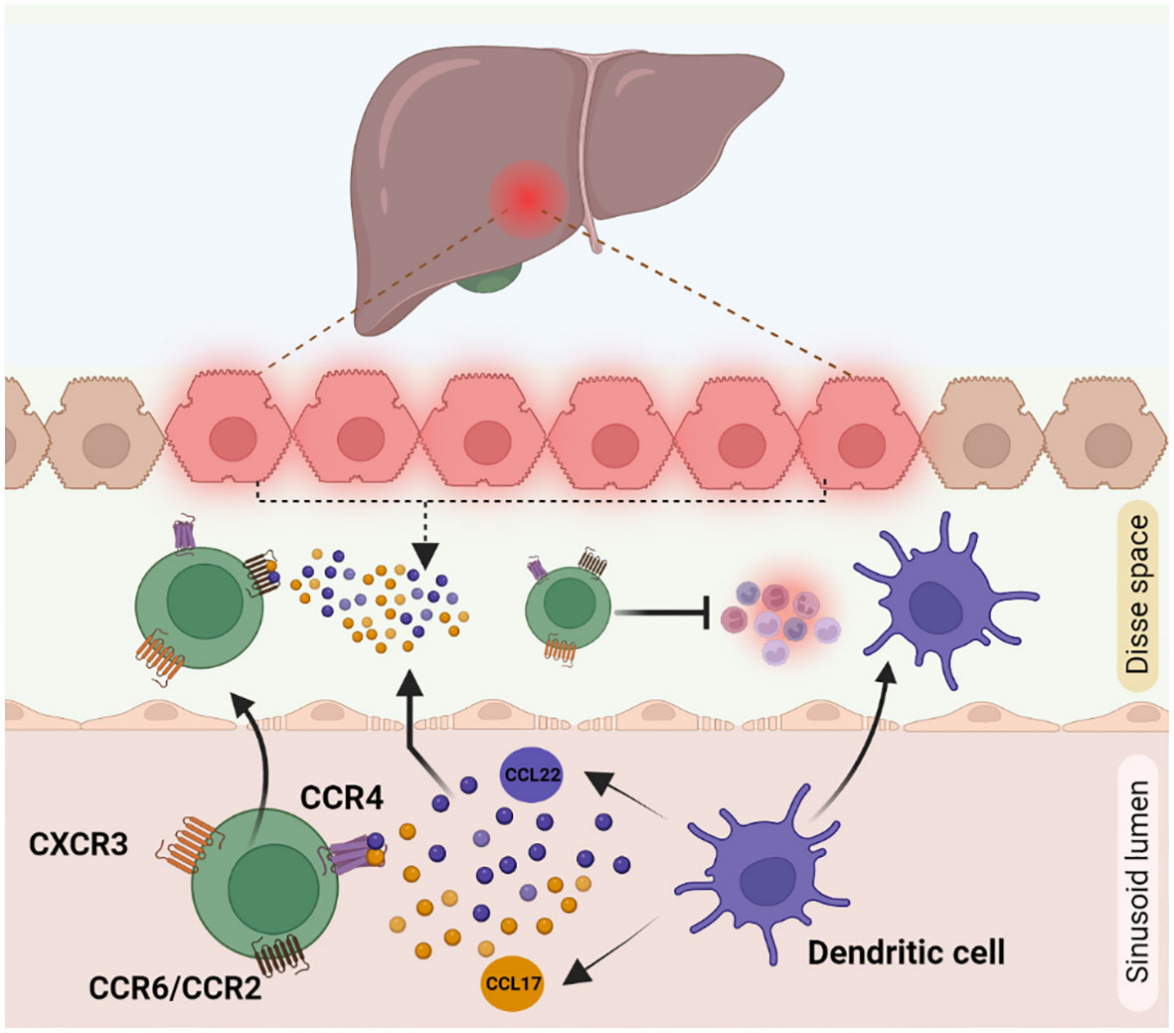
Cytokine mediated Treg recruitment during inflammation in liver: Treg recruitment towards the site of inflammation is required for other immune cell suppression during inflammation. This phenomenon is mainly mediated by receptors like CXCR3, CCR4 and CCR6 present on Treg cell surface. (1) CXCR3 helps Tregs in trans-endothelial migration across hepatic sinusoidal endothelium (2) CCR4 receptor, helps to respond to the chemokines CCL17 and CCL22 secreted by dendritic cells to co-localise with them to the inflamed liver. (3) CCL22 and CCL17 secretions by damaged hepatocytes are identified by CCR6/CCR2 receptors on Tregs, which helps in the active migration of Tregs.

Migration of immune cells to the site of inflammation is an important process, mediated by secreted cytokines and chemokines, for an effective immune response. The homing receptors expressed by Tregs are specific to the location and microenvironment at which they are exposed to, during their activation. Homing of Tregs in non-lymphoid tissue is mediated via higher expression levels of cell surface receptors like chemokine receptor 3 (CXCR3), CD103, C-C chemokine receptor type 5 (CCR5), CCR4, and CCR6. Recruitment of Tregs to the site of inflammation is facilitated by CXCR3, CCR5, and CCR6 receptors. Chemokine receptors CXCR3, CCR6 and CCR4 overlap with those of other effector T cells like Th1, CD8 T cells, Th17 cells and enable Tregs to co-localize with them (100). During Tregs infiltration, CXCR3 helps to undergo trans-endothelial migration across the hepatic sinusoidal endothelium. Naïve CXCR3 deficient mice displayed lower number of CD4^+^ CD25^+^ Tregs than in wild type mice. Accordingly, CXCR3 deficient mice tend to develop severe liver injury due to a lower infiltration of Tregs (101, 102). CCR4 helps Tregs to co-localize with liver infiltrating DCs by responding to chemokines CCL17 and CCL22 secreted by those cells (**Figure 3**). In chronic inflammatory liver diseases, liver infiltrating Tregs express both CXCR3 and CCR4. CCL17 and CCL22 secreted by activated intrahepatic DCs guide the CCR4 receptor in Tregs for the subsequent migration within the inflamed liver (103). Infected primary human hepatocytes secrete chemokines CCL20 and CCL22, which activate CCR6 and CCR2 present on Tregs thereby recruiting them to the site of inflammation (94). Crosstalk between the other liver immune cells and Tregs also regulates their infiltration into the inflammation site. The recruitment and the ability to maintain Tregs in the inflamed liver is influenced by several parameters, including intricate interactions with other extra-hepatic immune cell populations. Indeed, the role of GATA-Binding protein-6^+^ peritoneal macrophages in this complex orchestration has been recently unveiled, in the dual modulation of hepatic immunopathogenic genesis and the concurrent augmentation of Treg assemblages (104).

### Immune microenvironment and Tregs in HCC

**Figure 4:**
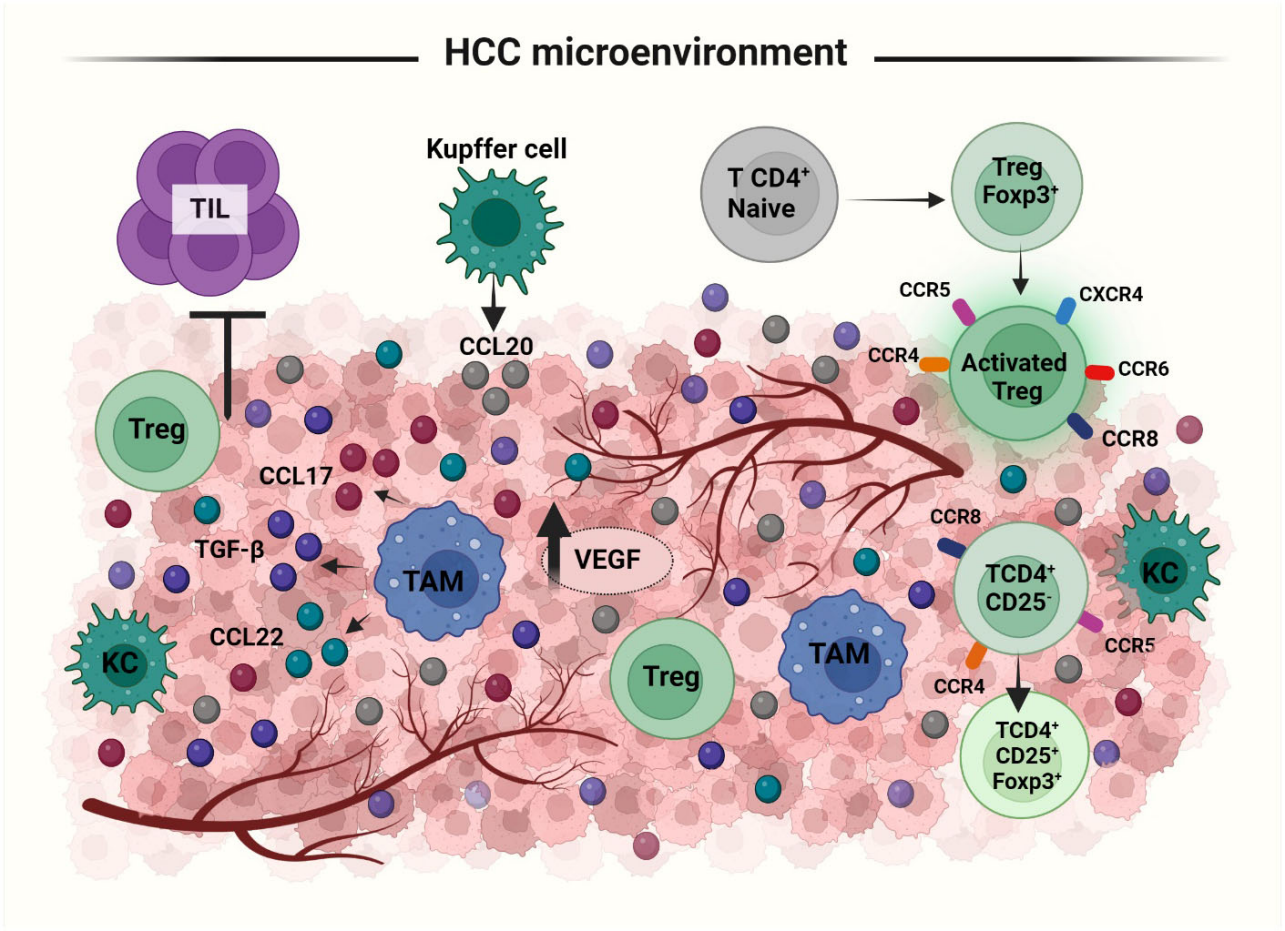
Recruitment and activation of Tregs to HCC tumor microenvironment (TME): Treg recruitment into TME happens by two mechanisms: priming of naïve T cells to differentiate into FoxP3 expressing Tregs or selective migration of Tregs by cytokines. In response to the cytokine secretion from TME, Tregs upregulate the expression of several receptors like, CXCR3, CCR5, CCR4, CCR8 etc. Treg migration is facilitated via the secretion of i) CCL20 by Kupffer and tumor cells, ii) CCL22 and CCL17 by tumor associated macrophages and iii) SDF1, TGFβ from tumor cells. TAMs; Tumor Associated Macrophages, KC; Kupffer Cells, TILs; Tumor Infiltrating Lymphocytes.

Cancer immune response is dependent on the balance between tumor antigenicity and the microenvironment of the tumor tissue. Immune cells like Th1 cells, Th2 cells, Tregs, myeloid derived suppressor cells, tumor associated macrophages, DCs, NKs, CD4^+^ and CD8^+^ T cells are all involved in the regulation of immune response in the tumor microenvironment (105). Tregs in the HCC microenvironment, have a suppressive activity. An expansion of CD4^+^ CD25^+^ T cell population is observed in both peripheral blood and tumor microenvironment of HCC patients (106, 107). An increased number of Tregs in tumor microenvironment can have an unfavorable outcome on immunoregulatory activities. Treg population has been shown to increase in HBV infected HCC patients (108). The two possible pathways for the recruitment of Tregs to the tumor microenvironment are either influenced by the priming of naive CD4^+^ T cells to differentiate into CD4^+^ CD25^+^ Tregs or by inducing the selective migration of Tregs via specific chemokine secretion (46) (**Figure 4**). In tumor microenvironment, CD4^+^ CD25^low^ T cells and CD4^+^ CD25^-^ T cells may get induced for FOXP3 expression and converted to CD4^+^ CD25^+^ Tregs. Tumor derived TGFβ plays an important role in this promotion of FoxP3 expressing Tregs from naïve CD4^+^ T cells. In mouse, the principal mechanism for increasing CD4^+^ CD25^+^ presenting FOXP3^+^ population in the tumor site is via the conversion of CD4^+^ CD25^-^ T cells (109, 110). Upregulation of chemokine receptors like CXCR3, CCR5, CCR4 and CCR8 is involved in both the activation and differentiation of Tregs in HCC. CCL22 produced by tumor derived macrophages and CCL17 regulate the infiltration of Tregs into the tumor sites. The CCR6-CCL20 axis plays an important role in recruiting Tregs to the tumor site. High levels of CCL20 are secreted by tumor cells and KCs to which CCR6 expressed in Tregs gets attracted inducing the migration towards the tumor site (46, 111). Secretion of the chemokine SDF-1 through the activation of CXCR4/CXCL12 signaling also has a positive correlation with increased Treg number in the tumor microenvironment (44). Thus, lack of proper tumor immunity is not due to the absence of immunogenicity but due to the increased infiltration and conversion of CD4^+^ CD25^-^ T cells to CD4^+^ CD25^+^ T cells in HCC.

### Clinicopathologic and prognostic significance of Tregs in HCC patients

The intra-tumoral balance between Tregs and cytotoxic T cells, determines the recurrence and survival rate of HCC. The number of Tregs in HCC microenvironment is proportional to the tumor size (112). Therefore, an increased incidence of CD4^+^ Tregs is associated with HCC invasiveness and poor prognosis (113). Higher grade of the tumor is correlated with both higher Treg and lower CD8^+^ T cell infiltrations (114). In the tumor microenvironment, CD4^+^ CD25^+^ Tregs have a negative impact on cytokine secretion and proliferation of CD4^+^ CD25^-^ anti-tumor T cells (44). CD8^+^ cells have been reported to accumulate in the peritumoral region, thereby proving that Tregs not only suppress CD8^+^ activity but also block their migration into the tumor. Besides inhibiting CD8^+^ T cell proliferation and activation, Tregs also suppress the production and release of the cytolytic enzymes perforin, granzyme A and granzyme B that mediate CD8^+^ T cell effector functions (109). Apart from CD4^+^ T cells not expressing CD25, Tregs were also documented for their ability to eliminate DCs, one of the most important surveillance mechanisms for the removal of cancer cells in the tumor microenvironment (115). Furthermore, the proportion of Tregs in the tumor microenvironment predicts HCC recurrence and survival rate. Indeed, the prevalence and migration of Tregs have been reported to enhance with the progression of HCC from an early to a later stage (44). A steady increase in the number of CD4^+^ has been noticed from hepatitis to liver cirrhosis to HCC (107, 111). HCC invasiveness is also associated with the number of Tregs. Indeed, HCC patients with lower intra-tumoral Tregs population had a higher overall survival and disease-free survival rates of 70 and 69 months when compared to patients with higher intra-tumoral Tregs population. Lower intra-tumoral Tregs with high CTL population in the HCC tumor are associated with an improved survival as well as a reduced recurrence in HCC (114). Analysis of specific surface markers of Tregs [GITR, HLA-DR, CD45RO, CD152, and CD45RA and FOXP3] also showed that there is a steady increase in the number of Tregs in both peripheral blood and tumor microenvironment (116).

Angiogenesis is another important feature of tumor progression and Tregs are known to play a positive role in HCC angiogenesis. increased levels of intra-tumoral FOXP3 cells was correlated to higher expression of vascular endothelial growth factor (VEGF) and microvascular density (117). HCC cells produce TGF-β1 which could generate Tregs from CD4^+^ CD25^-^ T cells. This tumor derived TGF-β1 negatively affects the tumor infiltrating leukocytes (TILs) thereby suppressing the immunosurveillance activity in tumor microenvironment. Another disadvantage correlated with TGF-β in HCC is that it induces epithelial-mesenchymal transition in HCC. Indeed, silencing TGF-β1 prolongs the survival rate in mouse models of HCC (1, 118). The levels of growth differentiation factor 15 (GDF15), which belongs to the family of TGF-β, are upregulated during inflammatory conditions or cancer as compared to normal physiological conditions. GDF15 promotes and modulates the proliferation of pTregs and the suppressive function of nTregs through CD48 T cell receptor and by post-transcriptional regulation of FoxP3 in humans (119). Constructing and validating an angiogenesis-related scoring (ARGs) model holds great promise for prognostication, tumor immune microenvironment assessment, and therapeutic stratification in HCC. A recent study harnessed the TCGA dataset to identify 97 differentially expressed ARGs linked to the prognosis of HCC patients. Nine-gene signature, accurately predicted unfavorable clinical outcomes, standing out as an independent prognostic indicator for HCC. Furthermore, associations with diverse immune cell enrichment, encompassing CD4^+^ T cells, Tregs, macrophages, neutrophils, and DCs have been raised, showcasing an inherently immunosuppressive phenotype. Accordingly, a higher ARGs Score reflects an elevated expression of immune checkpoint genes and an ineffective response to immunotherapy (120). In short, there are numerous regulatory mechanisms that initiate the activation and modulation of the immunosuppressive nature of Tregs resulting in the progression of HCC condition. Increasing evidences supported the involvement of new immune cell subtypes in orchestrating the recruitment of Tregs within the tumor microenvironment, particularly in HCC, including IFNγ- Tc17 cells, a distinct subset of CD8 cells producing IL-17. These cells display characteristics that foster tumor progression, marked by elevated CCL20 expression. Consequently, this triggered an escalation in the Tregs infiltration in the tumor microenvironment, correlating with an unfavorable prognosis (121). by using single-cell RNA sequencing (scRNA-seq) and flow cytometry, a specific subset of the innate-like mucosa-associated invariant T cells termed regulatory Mucosal associated invariant T cells (MAITregs) were detected within HCC patients. These MAITregs possess robust immunosuppressive abilities and are derived from a precursor reservoir exhibiting mild Treg-associated characteristics. Their development is triggered under Treg-promoting conditions like β1 adrenergic receptor signaling. Intriguingly, intra-tumoral MAITregs exert immune suppressive effects and are linked to unfavorable clinical outcomes in HCC patients (122).

## Clinical application of Tregs in liver diseases

Alteration of Tregs expression may have deleterious responses in tissues like i) suppression of effector immune response resulting in cancer progression ii) impaired autoimmune and inflammatory responses, and iii) protection against immune cells in viral infection. This suppressive population of immune cells has to be finely tuned to maintain the delicate balance between inflammatory insults, autoimmunity induction and facilitating protection against infection and tumor. Thus, selective purification and expansion of FOXP3^+^ Tregs is a potent cellular immunotherapy mechanism that could potentially treat T cell mediated inflammatory liver injuries and liver transplantation rejection. For successful Tregs-mediated cell therapy, strategies have been developed to maintain their phenotypic stability and suppressive capabilities post-infusion into the inflamed liver microenvironment. Neutralization of pro-inflammatory cytokines, IL-12 and IL-6 present in the inflamed intrahepatic microenvironment, can stabilize the Tregs (123). *In vitro*, the mTOR inhibitor rapamycin, in presence of TCR and CD28 or IL-2, can influence rapid expansion and proliferation of Tregs and increase their activity against effector T cells than Tregs grown in the absence of rapamycin (124). TX527, a vitamin D analogue, triggered the induction and migration of Tregs to the site of inflammation and induced the Tregs-based suppression of effector T cell functions. This Tregs’ immunosuppressive activity can be exploited for their therapeutic applicability in inflammatory and autoimmune disorders (125).

### Tregs immunotherapy for liver transplantation and autoimmune liver diseases

In the liver, crosstalk between leukocytes, hepatocytes, HSCs, LSECs, and cholangiocytes is important for maintaining the balance between immune reactivity and immune tolerance. For patient survival after liver transplantation, lifelong intake of immunosuppressants is a necessity. As an alternative approach, a small group of patients underwent Tregs-based therapy. Cells were generated *ex vivo* by co-culturing recipient lymphocytes with irradiated donor cells in the presence of anti-CD80/CD86 antibodies and were administrated back into the patients. The study was found to be a safer and an effective approach in maintaining immune tolerance without the intake of immunosuppressants (126), thereby proving the important role of Tregs in the induction and maintenance of immune tolerance after liver transplantation (127). During organ transplantation and due to graft-versus-host disease (GvHD), donor T cells get continuously activated and thus react with the host, resulting in tissue damage and extreme complications. Preclinical trials have demonstrated that adoptive transfer of Tregs can regulate GvHD (16). The first clinical trial in the treatment of acute and chronic GvHD using Tregs was performed in 2009. In chronic GvHD, reduction in symptoms and increase in the number of Tregs were observed while in acute GvHD, no significant conclusion was drawn (128).

A breakdown in immune homeostasis mediated by a balance between effector T cells and Tregs results in autoimmune liver diseases (AILDs) like AIH, primary biliary cholangitis and primary sclerosing cholangitis in liver. The current strategies of AILDs are not fully effective and require long term immunosuppressive medications leading to a poor control over hepatic and biliary inflammations. Tregs are highly sensitive to IL-2 which is required for Tregs survival (129). Trials on administration of low doses of IL-2 as an immunomodulatory agent into AIH patients showed positive therapeutic results (130). In animal models, gene transfer for the ectopic expression of autoantigens to liver cells has successfully used Tregs as a therapeutic option for inflammatory and autoimmune diseases. Selective delivery of autoantigens using nanoparticles to hepatic cells like LSECs helps in the generation of auto-antigen specific Tregs in the liver. Such approach has been proved to be effective at least in mice (131). In humans, Tregs therapy in autoimmune liver diseases (AILD) is not yet tested, but similar tests in Type 1 diabetes mellitus was found to be successful suggesting a possibility for AILD based Tregs therapy in the future (35).

### Immunotherapy targeting Tregs in HCC

HCC is the most prevalent form of primary liver cancer with various causes for tumorigenesis. Immunocompromised tumor environment due to natural suppressor cells like CD4^+^ CD25^+^ T cells is one of the major reasons for tumor progression and malignancy. TILs have a negative impact on the progression of solid tumors and a positive impact in anti-cancer therapies and prognosis of cancer (132). The two strategies employed for cancer immunotherapy aim at i) enhancing the number and function of immune effector cells and ii) blocking the immune suppressor cells like Tregs in the tumor microenvironment. Depletion and prevention of Tregs proliferation in the tumor microenvironment, represent accordingly a potential therapeutic measurement for HCC (133). In a mouse model of HCC, treatment with anti-CD25 antibody [PC-61] resulted in the suppression of Tregs thereby reducing tumor growth. Treatment with antibody for chemokine receptor CCR4 reduced the intra-tumoral population of Tregs as well as the tumor growth (**Table 3**) (134–136). Furthermore, targeting OX40, an immune checkpoint extensively expressed in Tregs, could result in the depletion of HCC tumor cells. A combination therapy using anti PD-1 antibody Nivolumab and anti CTLA-4 antibody ipilimumab resulted in tumor remission in 29% of patients with HCC within 6 weeks of treatment (**Table 3**) (133, 137). Sirolimus and sorafenib induce apoptosis of Tregs when activated through T cell receptors. Therefore, these molecules can be used to reduce the tumor recurrence rate by keeping the Tregs population regulated (**Table 3**) (112). A potential mechanism underlying the increased intra-tumoral Treg accumulation induced by anti-PD-1 treatment in HCC, has been proposed. By using single-cell transcriptomic approach, the study reveals that Nrp-1 facilitates the migration behavior of Tregs, and the genes Crem and Tnfrsf9 governing the activity of the terminal suppressive Tregs. As Tregs transition from lymphoid tissues to the tumor, a progression from Nrp-1 + 4-1BB^-^ Tregs to Nrp-1^-^ 4-1BB^+^ Tregs was reported. Depleting Nrp-1 specifically in Tregs curtailed the heightened intra-tumoral Tregs resulting from anti-PD-1 therapy, and its combination with a 4-1BB agonist enhances the antitumor response. Importantly, employing a Nrp-1 inhibitor alongside a 4-1BB agonist in humanized HCC models demonstrates favorable outcomes and safety, augmenting the anti-tumor effects of PD-1 blockade (6). Administration of daclizumab, a blocking antibody that reduces the Treg numbers, is a potential immunotherapy option for HCC. Depleting the local Treg population while preserving the host immune response, is therefore an important strategic approach for successful immunotherapy in HCC (138).

**Table 3:** Treg based immunotherapy trials in HCC.

Treatment	Model	Regulation	Disease condition	Reference
PC-61 (anti-CD25 Ab)	Mouse	Downregulated	HCC	(134)
CCR4	Mouse	Downregulated	HCC	(135)
Combination of Nivolumab (anti-PD-1) & ipilimumab (anti-CTLA-4)	Human	Downregulated	HCC	(133)
Sirolimus, sorafenib	Human	Tregs apoptosis	HCC	(112)

Notably, Tregs have a detrimental effect in HCC malignancy due to their anti-tumor immune cell suppression. Thus, immunotherapy targeting the Tregs is an effective way for preventing the progression of non-cancerous lesions into HCC. In this context, focusing on miRNAs to decrease Tregs is an alternative strategy. Recent research illustrates the efficacy of miR-22 delivery via adeno-associated virus in treating HCC, showing superior survival outcomes and lower toxicity when compared to FDA-approved lenvatinib. In liver, miR-22 reduces IL17-producing T cells, resulting in the attenuation of IL17 signaling via HIF1α. Moreover, miR-22 fosters the expansion of cytotoxic T cells while concurrently reducing the presence of Tregs (139).

### Generation of GMP-compliant clinical grade Tregs for cell therapy

Adoptive Tregs therapy is an alternative approach for harnessing a Tregs-induced immune suppressive activity. For this approach, Tregs are isolated from the patient, expanded *in vitro* and reinfused back to the patient. The advantage of this approach is that, the phenotype and functionality of the Tregs can be analyzed prior to their administration (140). Cell surface markers CD4, CD25^high^ CD127^low^ help in the identification of Tregs from non-Tregs (141). However, the functional instability of Tregs pose a challenge for adoptive Tregs therapy, as there is a chance for these infused Tregs to lose FOXP3 expression due to the influence of pro-inflammatory factors and to shift towards effector T phenotypes secreting IL-17 and interferon-γ (48). Firstly, Peripheral blood mononuclear cells (PBMCs) isolated from peripheral blood are subjected to density gradient centrifugation. Via CliniMACS selection, PBMCs were isolated and subjected to negative selection of CD4^+^ using antibodies against CD8, CD14, CD16, CD19, CD33, CD238a and a positive selection using anti-CD25 antibody for obtaining a high purity CD4^+^ CD25^+^ T cell fraction. For expansion and selective enrichment for CD25^+^, T cells were activated with anti-CD3/CD28 beads and occasionally re-stimulated with rapamycin, IL-2 and RA. During harvest of the expanded Tregs, IL-17 and IFNγ expression is analyzed by intracellular staining and indirect sandwich ELISA methods to deplete CD127^+^ cells and selectively culture CD4^+^ CD25^+^ T cells (142). The suppressive capacity of cultured Tregs is studied in co-culture assays (143). In phase I clinical trial conducted at King’s College Hospital London and University Hospitals Plymouth (UK), Tregs expanded under Good Manufacturing Practices (GMP), were administrated back into the patient at two dosages of 0.5-1 million Tregs/kg and 3-4.5 million Tregs/kg. The study showed that in patients administered with 4.5 million Tregs/kg, the transfer increased the circulating Tregs pool for 1 month as compared to the Treg pool before infusion (144). Cell tracking and dose escalating studies to monitor the safety and efficacy of Tregs are necessary to confirm the accumulation of Tregs in the site of inflammation. However, Tregs choice and antigen specificity, adequate cell number, timing and frequency of administration are still to be considered for achieving adoptive cell therapy (123).

### Engineering specificity and function of therapeutic Tregs

The major disadvantage of adoptive Treg cell therapy is that, Tregs fail to maintain their phenotypic characters for immunosuppression at longer time periods. To overcome this shortcoming, a vector encoding TCR or chimeric antigen receptor (CAR) with defined antigen specificity Tregs, was developed. TCR expressed on Tregs surface determines their specificity. Tregs encoding the genes with TCR or CAR can be genetically engineered to increase the Tregs specificity to antigens present during autoimmunity and not in normal cells. CARs have MHC independent recognition, so they are applicable to patients irrespective of their genotypes (145 *)*. CARs have an extracellular antigen recognition site, transmembrane domain and hinge site connected to the intracellular signaling domain. CAR based mechanism of suppression is similar to Tregs mediated immune cell suppression. Suppression of DCs is accomplished by binding to its CD80/CD86 cell surface protein. It can interfere with the metabolism of lymphocytes, via the secretion of anti-inflammatory cytokines like IL-10, IL-35 and TGFβ or direct cell-to-cell interaction induced inhibition and apoptosis (146). Delivery of CAR is by viral vectors like lentiviruses, adeno-associated viruses or adenoviruses or it can be through non-viral vectors like transposons or plasmid vectors (147). Using nanocarriers as vectors can be a safer option than conventional viral and non-viral vectors. The next step in Tregs engineering can be using newer techniques as clustered regularly interspaced short palindromic repeats (CRISPR) by removing genes for cytokine signals like IL-6, TNFγ, and IL-17. Preclinical trials in immunosuppressive diseases have been successful using CAR expressing Tregs (148). These encouraging findings have paved the way for the first clinical trial involving CAR Tregs sponsored by Sangamo Therapeutics, authorized by the UK MHRA and the US (NCT04817774), and targeting kidney transplanted patients. Several other biopharmaceutical companies are closely pursuing similar initiatives, with Quell Therapeutics focusing on CAR Tregs for liver transplanted recipients, indicative of the growing interest and potential in this field (149). An additional study involving HLA-A2-specific CAR Tregs, termed LIBERATE (Phase I/II), is started in 2022 and its primary completion estimated in 2025. Notably, this study will uniquely investigate both clinical outcomes and immunosuppression in the context of liver transplantation (QEL-001). This forward-looking initiative aims to provide valuable insights into the potential efficacy and broader implications of CAR Treg therapy in the realm of liver transplantation (150).

During liver transplantation and autoimmune liver diseases, Tregs are important to prevent GvHD and self-cell destruction. Thus, the future prospects would be the development and administration of Tregs *in-vitro* cultured or genetically engineered for expressing specific antigens required for self-cell destruction by other immune cells. While, during HCC progression, it is necessary that the Treg numbers are under control for the cytotoxic T cell population to eliminate tumor cells. To summarize, the dysregulation of Tregs is deleterious on the liver disease.

## Conclusion

Tregs play an important role in the monitoring of immune balance and have a protective role against excessive inflammation. Tregs are heterogenous cell populations differentiated based on their site of localization, cell markers, homing receptors and chemokine secretions. Tregs play different roles in chronic liver diseases and their progression towards HCC. During liver fibrosis, Tregs have a protective role in preventing chronic inflammation, but in liver tumor microenvironment, the increase in Tregs’ numbers can be detrimental because Tregs target the T effector and NK cells which are required for killing tumor cells and preventing tumor progression and metastasis. Thus, it could be concluded that Tregs play an important role in the progression of chronic liver diseases toward carcinogenesis. Also due to the recent advances in the field of immunotherapy, studies are focusing on using these cells in preventing autoimmune diseases, while using specific blockers to inhibit their activity or altering their phenotype to reduce the immunosuppressive properties in tumor microenvironment. Thus, deeply understanding the mechanism of Tregs behavior in chronic liver diseases can be useful for elucidating the therapeutic options to adopt for preventing the progression of the chronic liver disease to HCC.

## Author contributions

AA: Writing – review & editing, Writing – original draft. MM: Writing – review & editing. MA: Writing – review & editing. NH: Writing – review & editing. MeN: Writing – review & editing. MV: Writing – review & editing. ES: Writing – review & editing. MN: Writing – review & editing.
